# Can Arthrogenic Muscle Inhibition Exist in Peroneal Muscles Among People with Chronic Ankle Instability? A Cross-sectional Study

**DOI:** 10.1186/s40798-024-00710-y

**Published:** 2024-04-10

**Authors:** Shiyu Dong, Yanhao Liu, Ziyin Liu, Peixin Shen, Hao Sun, Ping Zhang, Daniel T.P. Fong, Qipeng Song

**Affiliations:** 1 College of Sports and Health, Shandong Sport University, Jinan, 250102 Shandong China; 2 National Centre for Sport and Exercise Medicine, Loughborough University, Loughborough, UK

**Keywords:** Ankle sprains, Central activation ratio, Superimposed burst technique, Motor neurons

## Abstract

**Background:**

Ankle sprains lead to an unexplained reduction of ankle eversion strength, and arthrogenic muscle inhibition (AMI) in peroneal muscles is considered one of the underlying causes. This study aimed to observe the presence of AMI in peroneal muscles among people with chronic ankle instability (CAI).

**Methods:**

Sixty-three people with CAI and another sixty-three without CAI conducted maximal voluntary isometric contraction (MVIC) and superimposed burst (SIB) tests during ankle eversion, then fifteen people with CAI and fifteen without CAI were randomly invited to repeat the same tests to calculate the test-retest reliability. Electrical stimulation was applied to the peroneal muscles while the participants were performing MVIC, and the central activation ratio (CAR) was obtained by dividing MVIC torque by the sum of MVIC and SIB torques, representing the degree of AMI.

**Results:**

The intra-class correlation coefficients were 0.77 (0.45–0.92) and 0.92 (0.79–0.97) for the affected and unaffected limbs among people with CAI, and 0.97 (0.91–0.99) and 0.93 (0.82–0.97) for the controlled affected and unaffected limbs among people without CAI; Significant group × limb interaction was detected in the peroneal CAR (*p* = 0.008). The CARs were lower among people with CAI in the affected and unaffected limbs, compared with those without CAI (affected limb = 82.54 ± 9.46%, controlled affected limb = 94.64 ± 6.37%, *p* < 0.001; unaffected limb = 89.21 ± 8.04%, controlled unaffected limb = 94.93 ± 6.01%, *p* = 0.016). The CARs in the affected limbs were lower than those in the unaffected limbs among people with CAI (*p* = 0.023). No differences between limbs were found for CAR in the people without CAI (*p* = 0.10).

**Conclusions:**

Bilateral AMI of peroneal muscles is observed among people with CAI. Their affected limbs have higher levels of AMI than the unaffected limbs.

## Key Points

By using the SIB technique, AMI was observed bilaterally in peroneal muscles among people with unilateral CAI.

The affected limbs have higher levels of AMI compared to the unaffected limbs among people with CAI.

Bilateral peroneal strength rehabilitation should be performed among people with unilateral CAI to reduce the effects of AMI on bilateral peroneal strength.

## Background

Ankle sprains are one of the most common musculoskeletal injuries [1, 2], accounting for approximately 15% of sports-related injuries [3], with a recurrence rate as high as 80% [4]. Up to 40% of people with acute ankle sprains may develop into chronic ankle instability (CAI) [5], characterized by pain, subjective instability, and re-sprains [6] due to mechanical laxity or functional deficit of ligaments following acute ankle sprains. Long-term dysfunction and high recurrence rates have perplexed clinical professionals to examine the causes of CAI. Lack of understanding of the neurophysiological mechanisms precludes the development of effective CAI rehabilitation programs.

The decreased strength of the peroneal muscles is a critical factor in recurrent ankle sprains among people with CAI [7, 8]. Peroneal muscles are the primary muscles to prevent excessive ankle inversion [9], in which most ankle sprains occur [1]. However, people with CAI have decreased ankle eversion strength, leading to recurrent ankle sprains [9, 10], which can further reduce peroneal muscle strength [11], triggering future sprains. Moreover, persistent peroneal muscle weakness may decrease ankle stability [8] and lead to osteoarthritis [12].

To the best of our knowledge, the neurophysiological mechanisms of peroneal muscle weakness are still unknown, and arthrogenic muscle inhibition (AMI) may potentially be one of the causes [13]. AMI represents a protective mechanism triggered by the injury to the periarticular muscles or joint structures that protects the muscles from increased stress after injuries [14, 15]. It diminishes the efficiency of motoneuron recruitment and the autonomous activation ability of muscles, by activating inhibitory interneurons synapsing in the motor neuron pool [16, 17]. The activation of inhibitory interneurons reduces the recruitment efficiency of the motor neuron pool [15, 18], thereby decreasing the strength of muscles and increasing the risk of ankle sprains. In addition, the activation time of the peroneal muscles during walking was earlier and prolonged among people with CAI than those without CAI [19], suggesting they have different neuromuscular activation strategies.

Inhibition of motor neuron activation among people with CAI has been observed by the H-reflex [13], a spinal monosynaptic reflex with a pathway that runs from Ia afferent fibers to α motoneurons [20, 21]. However, the decrease of H-reflex amplitude has weakness to prove the presence of AMI. (1) The H reflex amplitude is obtained by calculating the ratio of H- and M- wave amplitudes while stimulating the tibial nerve in the popliteal fossa, represents the capable of activation and the entire activation of the moto neuron pool [22], respectively. The representativeness of the M-wave as motor neuron pool activation has been challenged [23]. (2) The amplitude of the H-reflex can be affected by external factors, such as electrode placement, head and body posture, foot position, and even eye movement [24–26]. (3) The H-reflex is often elicited by a square wave pulse of about 0.5-1ms, triggering a muscle response (EMG, not force) within 100ms [27]. The muscle contraction force deficits caused by motor neuron inhibition cannot be observed through it. The superimposed burst (SIB) technique could be used to directly confirm the presence of AMI by showing the muscle force decrease caused by AMI. It applies electrical stimulation to the muscles while the participant performs maximal voluntary isometric contraction (MVIC), and the peak torques during MVIC and after stimulation are referred to as the MVIC and SIB torques [28]. The central activation ratio (CAR) can be obtained by dividing MVIC torque by the sum of MVIC and SIB torques, representing the ratio of possible voluntary muscle contraction force and the entire muscle contraction force. A CAR < 95% usually indicates the presence of AMI [26]. The SIB technique has been used to detect AMI in the quadriceps after knee injuries [29, 30], however, to our knowledge, no studies have yet confirmed the presence of AMI in peroneal muscles among people with CAI during isometric ankle eversion using SIB technique.

This study aims to observe the presence of AMI in peroneal muscles among people with CAI by SIB technique. We compared the CARs between the people with and without CAI and within their affected and unaffected limbs. Our hypotheses were as follows: (1) CARs were lower among people with CAI than those without CAI, and (2) CARs were lower in the affected limbs than in the unaffected limbs among people with CAI.

## Methods

### Participants

The sample size was estimated based on a previous study that reported quadriceps CAR after anterior cruciate ligament reconstruction, in which the effect size (Cohen’s *d*) was calculated as 0.47 [31]. A minimum sample size of 43 in each group was required to achieve a statistical power of 0.80 and an α level of 0.05.

A total of 63 people with unilateral CAI (female = 13, male = 50, age: 21.3 ± 1.5 years, height: 176.9 ± 8.5 cm, mass: 70.8 ± 12.3 kg, 56 and 48 of them experienced swelling and pain after sprains) and another 63 without CAI (female = 13, male = 50, age: 22.5 ± 2.2 years, height: 174.2 ± 7.7 cm, mass: 67.1 ± 11.2 kg) participated in this study. The inclusion criteria followed the recommendations of the International Ankle Consortium [32]: (1) a history of at least one significant ankle sprain occurring one year before the tests, (2) a sensation of ankle joint instability (i.e., “giving way”) or recurrent sprains, and (3) a score less than 24 on the Cumberland Ankle Instability Tool. Exclusion criteria were: (1) a history of lower extremity fracture or surgery, (2) acute symptoms of an ankle injury, and (3) bilateral CAI. The inclusion criteria for people without CAI were no history of ankle sprains, and the exclusion criteria were consistent with those with CAI.

People without CAI were matched with people with CAI based on sex, age, height, and mass. To limit the potential bias of limb dominance (dominant, non-dominant), we counted the affected limb among people with CAI, with fifty-five people with CAI in the dominant limb and eight in the non-dominant limb. Thus, there were fifty-five dominant limbs and eight non-dominant limbs were matched as the controlled affected limb among people without CAI. The same was true for the other limb. We defined the dominant limb as the limb on which a ball is kicked. Before being enrolled, all participants gave informed consent, and the study was approved from the Institutional Review Boards of Shandong Sport University (2022001).

### Protocol

Sixty-three people with CAI and another sixty-three people without CAI completed the MVIC and AMI tests in their affected and unaffected limbs. After the test, fifteen people with CAI and another fifteen without CAI were randomly invited to perform the same tests one more time to calculate test-retest reliability. During the tests, the room temperature was set to 24℃ and humidity to 65%.

#### Maximal Voluntary Isometric Contraction Test

**Fig. 1:**
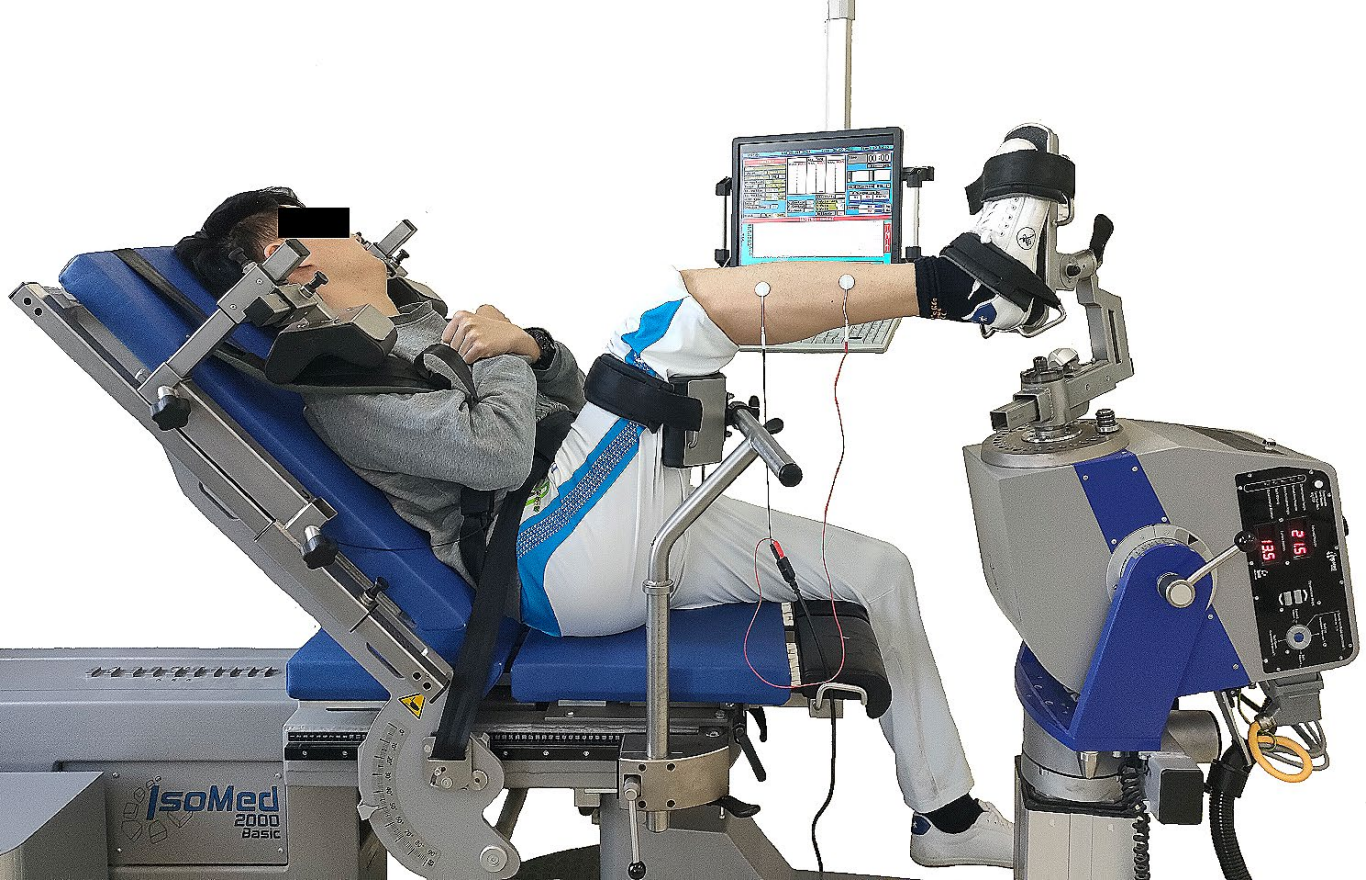
Illustration of superimposed-burst test in peroneal muscles

The ankle eversion MVIC test was conducted using an isokinetic dynamometer (IsoMed 2000, D & R Ferstl GmbH, Hemau, Germany). The participants were seated on the dynamometer seat, with their hip angle at 80° and knee angle at 110°, ensuring the shin was positioned parallel to the ground [33]. The distances between the chair and the foot adapter could be modified, along with the height of the adapter supported on the thigh. The foot was securely placed on the adapter, with the ankle at 10° of plantarflexion and 40° of inversion [34]. Two Velcro straps were used to stabilize the foot. Shoulder straps and pads were utilized to stabilize the shoulders, and the participant’s arms were crossed in front of their chests (Fig. 1).

Before the formal tests, participants conducted several MVICs to ensure they were familiar with the testing process. During the tests, participants performed two submaximal contractions at approximately 50% and 75% of their perceived maximal effort as a warm-up, followed by the three MVIC trials. The testers provided consistent verbal encouragement, such as “holding on” or “trying harder,” until the participants reached a torque plateau for at least 2 s. Participants were given a rest of 2 min between each set of MVIC trials to avoid fatigue.

#### Arthrogenic Muscle Inhibition Test

A constant-current electrical stimulator (Digitimer DS7AH, Digitimer Ltd., Hertfordshire, U.K.) delivered electrical stimulations to peroneal muscles. Before the test, the participant’s skin was cleaned with 75% alcohol wipes, and 2 mm diameter self-adhesive electrodes were applied to the belly of the peroneus longus (negative pole) and peroneus brevis (positive pole). An automated torque-triggering approach was employed, utilizing a custom-written LabVIEW program (LabVIEW 11.0; National Instruments Corp., Austin, TX, U.S.A.) to deliver the electrical stimulation. Briefly, three ankle eversion MVIC trials were done as described before, and the peak torque of the three trials was recorded. Suppose the following MVIC torque exceeds the peak torque, the LabVIEW program automatically triggers the electrical stimulator to deliver a train of three square-wave pulses of electrical stimulation at 100 Hz, with a width of 200µs and a current of 200mA [29, 35] (Fig. 1).

### Variables

**Fig. 2:**
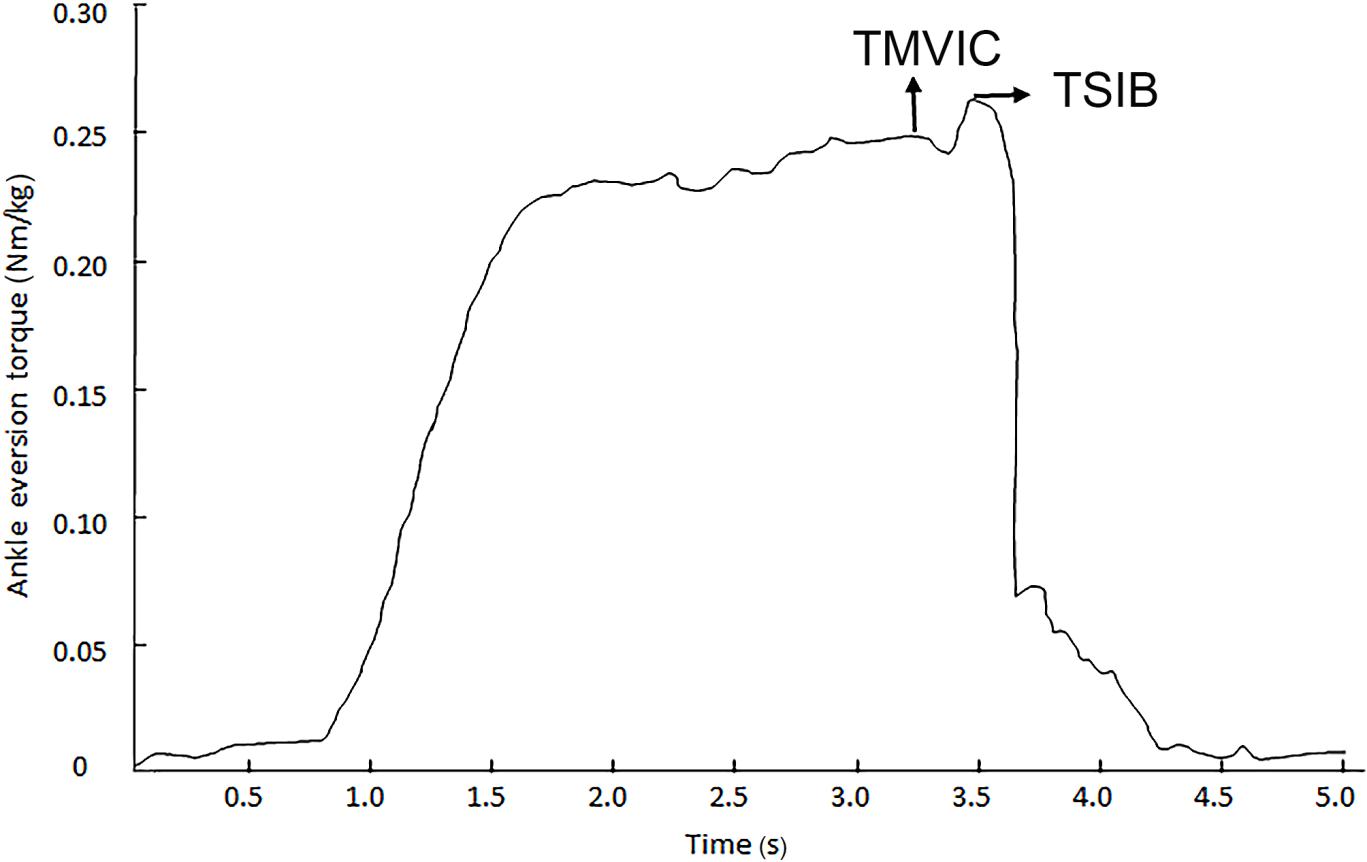
Illustration of calculation of the CAR. CAR: central activation ratio, TMVIC: maximal voluntary isometric contraction torque, TSIB: superimposed burst torque

All test data were recorded in Microsoft Excel. The average MVIC torque 200-millisecond before the stimulation is referred to as the MVIC torque, and the peak torque after stimulation is referred to as the SIB torque (Fig. 2). The CAR can be obtained by dividing MVIC torque by the sum of MVIC and SIB torques by computer, the calculation formula as follows:

$$CAR=\frac{TMVIC}{\left(TMVIC+TSIB\right)}\times 100\%$$

### Statistical Analysis

Statistical analysis was performed using SPSS software (version 26.0, IBM., U.S.A.). All outcomes were reported as means ± standard deviations. The test-retest reliability was determined using intra-class correlation coefficients (ICC) with a two-way mixed model. The 95% confidence interval (CI) of ICC was calculated. Thresholds of ICC were as follows: 0.0 to 0.5, poor; 0.5 to 0.75, moderate; 0.75 to 0.9, good; and 0.9 to 1.0, excellent [36]. Shapiro-Wilk tests were adopted to verify the normality of the outcomes. Two-way ANOVAs with repeated measures were used to detect the group (CAI vs. non-CAI) × limb (affected vs. unaffected) interaction and their main effects of CAR. Bonferroni adjusted independent or paired sample t-tests were used to determine the difference between the people with and without CAI, and between affected and unaffected limbs within the same group of people, respectively. The statistical significance was set as 0.05.

## Results

The test-retest reliabilities of CAR in peroneal muscles were assessed in 15 people with CAI and another 15 people without CAI (Table 1). The ICCs were 0.77 (0.45–0.92) and 0.92 (0.79–0.97) for the affected and unaffected limbs among people with CAI, and 0.97 (0.91–0.99) and 0.93 (0.82–0.97) for the controlled affected and unaffected limbs among people without CAI (Table 1).

**Table 1:** Test-retest reliability of the CARs in peroneal muscles among people with and without CAI

With CAI					Without CAI			
No.	Affected limb		Unaffected limb		Controlled Affected limb		Controlled Unaffected limb	
	Test	Re-test	Test	Re-test	Test	Re-test	Test	Re-test
PN01	70.59%	71.78%	72.73%	74.95%	95.68%	94.65%	94.12%	94.00%
PN02	73.92%	72.43%	78.57%	80.00%	96.37%	96.65%	94.74%	95.32%
PN03	66.67%	75.00%	85.71%	87.50%	99.25%	99.84%	99.77%	98.55%
PN04	96.30%	93.33%	96.00%	95.00%	96.48%	95.93%	95.45%	94.32%
PN05	91.67%	81.25%	94.74%	93.75%	80.45%	81.67%	86.43%	85.75%
PN06	92.31%	86.67%	92.00%	92.00%	97.63%	98.34%	96.55%	98.36%
PN07	88.89%	88.24%	92.86%	93.33%	91.96%	92.07%	92.86%	90.56%
PN08	85.71%	87.50%	89.47%	90.91%	86.86%	90.91%	90.31%	87.29%
PN09	80.92%	82.33%	78.57%	81.67%	99.64%	98.14%	98.83%	99.66%
PN10	80.00%	78.95%	86.67%	94.74%	94.26%	94.05%	95.65%	94.37%
PN11	93.75%	88.24%	91.67%	85.71%	95.81%	96.54%	97.74%	96.69%
PN12	77.78%	78.57%	92.31%	93.33%	97.53%	96.71%	96.00%	94.82%
PN13	73.24%	70.67%	72.33%	75.00%	80.49%	81.07%	80.33%	82.85%
PN14	76.92%	86.67%	92.86%	94.12%	98.75%	99.24%	95.24%	98.07%
PN15	96.15%	85.71%	96.30%	95.24%	98.65%	96.27%	98.65%	95.98%
Mean	82.98%	81.82%	87.51%	88.48%	93.98%	94.13%	94.17%	93.77%
S.D.	9.80	7.01	8.17	7.28	6.37	5.73	5.12	5.00
ICC	0.77		0.92		0.97		0.93	
95% CI	0.45–0.92		0.79–0.97		0.91–0.99		0.82–0.97	

CAR: central activation ratio, CAI: chronic ankle instability, PN: participant, S.D.:standard deviations, ICC: intraclass correlation coefficient, CI: confidence interval

**Fig. 3:**
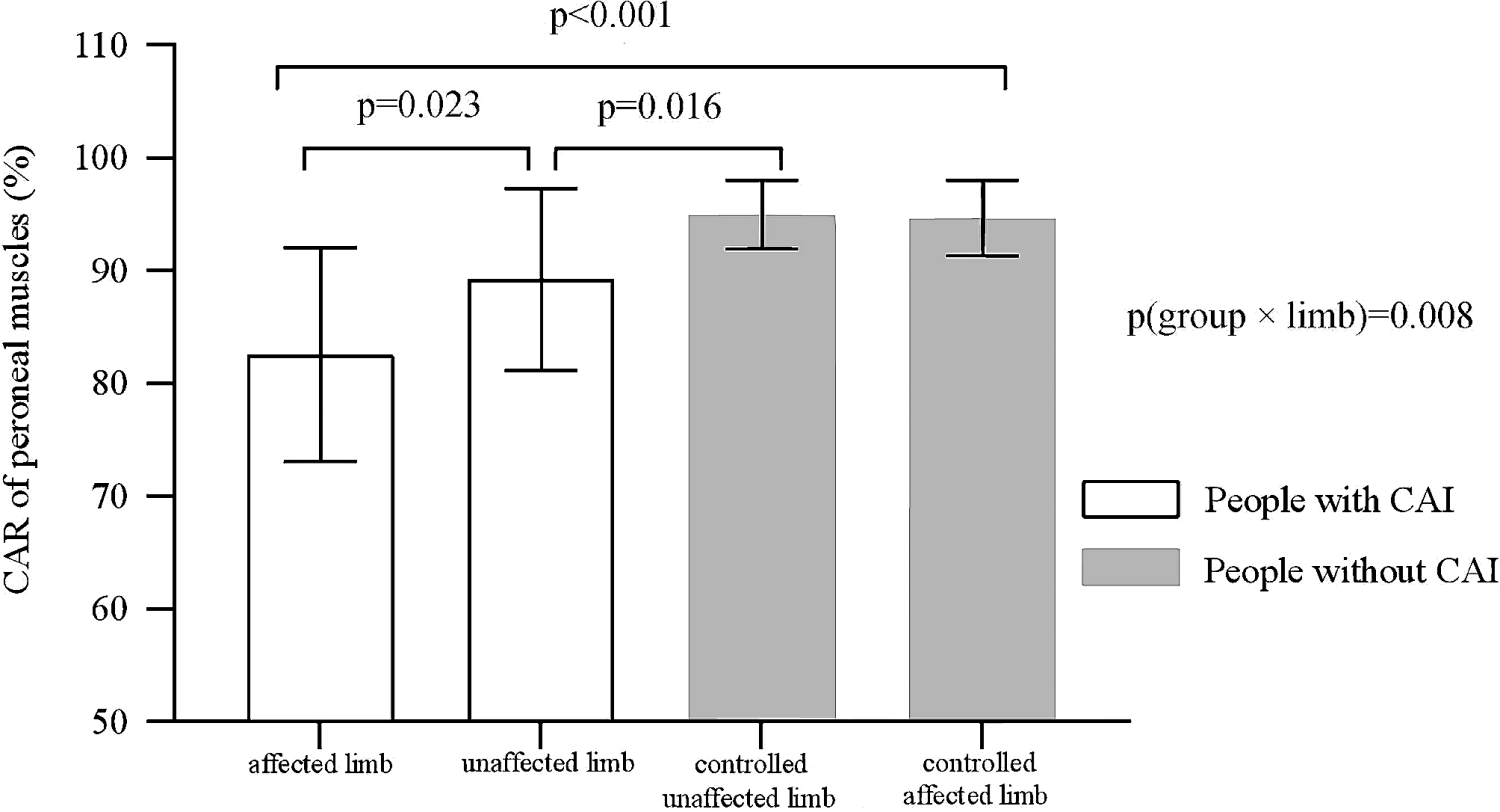
The CAR in peroneal muscles among people with and without CAI. CAI: chronic ankle instability, CAR: central activation ratio

Significant group × limb interaction was detected in CAR (*p* = 0.008). The CARs were lower among people with CAI in the affected and unaffected limbs compared with those without CAI. (affected limb = 82.54 ± 9.46%, controlled affected limb = 94.64 ± 6.37%, *p* < 0.001; unaffected limb = 89.21 ± 8.04%, controlled unaffected limb = 94.93 ± 6.01%, *p* = 0.016). The CARs in the affected limbs were lower than those in the unaffected limbs among people with CAI (affected limb = 82.54 ± 9.46%, unaffected limb = 89.21 ± 8.04%, *p* = 0.023) (Fig. 3). No differences between limbs were found for CAR in the people without CAI (*p* = 0.10).

**Fig. 4:**
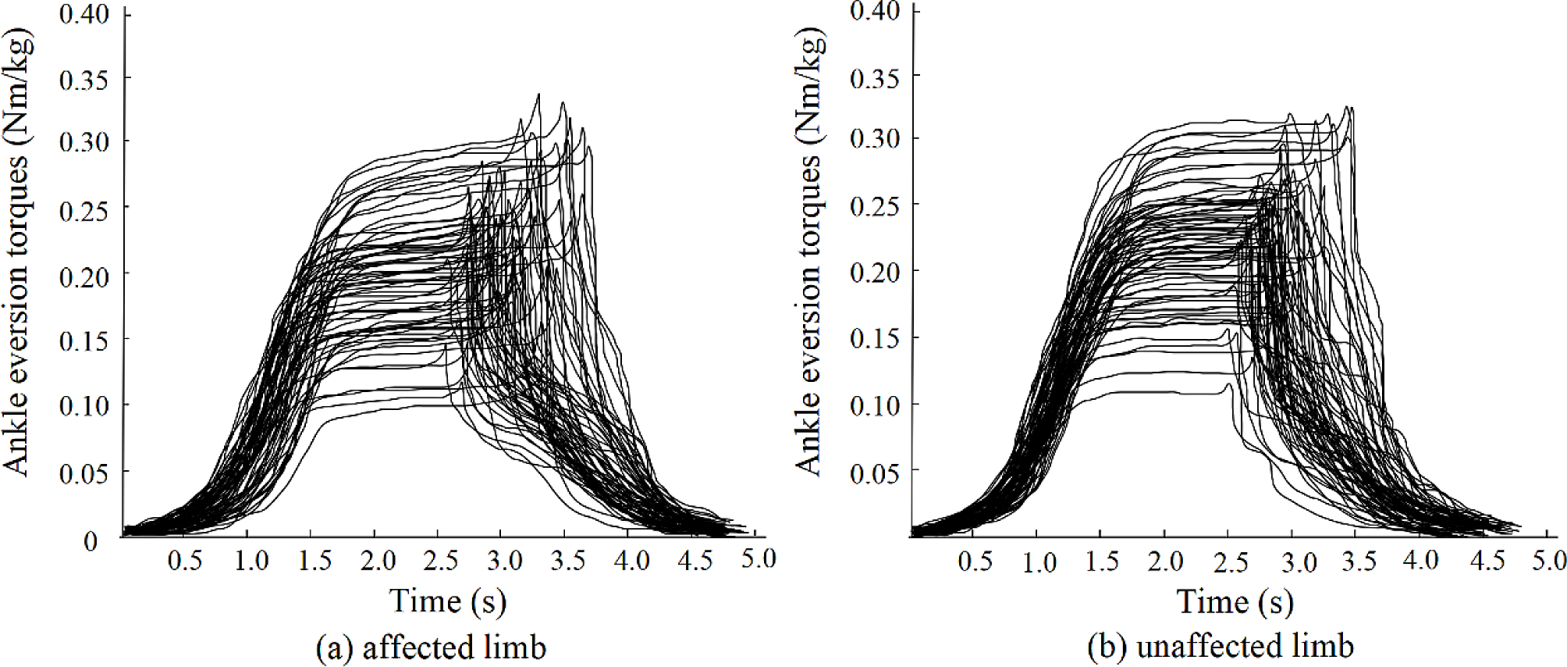
Eversion torques of the affected and unaffected limbs among people with CAI during AMI tests. CAI: chronic ankle instability, AMI: arthrogenic muscle inhibition

The ankle eversion torque curves in the affected and unaffected limbs for each participant among 63 people with CAI are shown in Fig. 4. The affected limbs exhibit relatively higher SIBs than the unaffected limbs.

## Discussion

This study observed AMI of peroneal muscles during isomeric ankle eversion among people with CAI. These findings suggest that AMI may be a contributing factor to the decreased muscle strength among people with CAI. The outcomes supported the two hypotheses: (1) CARs were lower among people with CAI compared to those without CAI, and (2) CARs were lower in the affected limbs compared to those in the unaffected limbs among people with CAI.

The SIB technique used to measure CAR in peroneal muscles demonstrated good to excellent reliabilities among people with and without CAI. These results align closely with previous studies that highlight the reliability of SIB technique in evaluating quadriceps CAR [37, 38]. Therefore, our findings indicate that SIB technique is reliable in measuring CAR in peroneal muscles during isometric ankle eversion. It needs to be noted that the ICC of CAR in the affected limb was lower than that in the unaffected limb, which may line in the dysfunction of the affected limbs among people with CAI. People with CAI usually have pain in the affected limb after ankle sprains [39], and the degree of pain in the ankle may be different between the two test sessions, leading to inconsistency of peroneal CAR. Moreover, people with CAI have peroneal neuromuscular control deficits [40], which may result in inconsistent peroneal muscle strength between the two test sessions.

Our study observed the presence of AMI in the affected peroneal muscles among people with CAI compared to those without CAI, which is supported by a previous study that showed the presence of AMI in the affected quadriceps after knee injury [29]. The observed AMI may be attributed to the symptoms experienced after ankle sprains. The altered afferent signals induced by swelling and pain have been shown to inhibit the activation of motor neurons. Swelling affects the recruitment of type Ib and type II afferent fibers, inhibiting alpha motor neurons within the spinal cord and decreasing motor output from the muscles in the affected limbs [14, 41]. Pain also leads to similar inhibition on alpha motor neurons owing to the activation of type III and type IV afferent nociceptors [42, 43].

Moreover, we observed the presence of AMI in the unaffected limbs among people with CAI. Similar bilateral activation defects have been reported after knee injuries, which limit the bilateral strength of unilateral injuries [38]. These bilateral activation defects may be attributed to a “cross-over” effect in the central nervous system. Corticospinal tracts emanating from the brain’s primary motor cortex cross over in the medulla oblongata and eventually establish synaptic connections with contralateral spinal motor neurons to control contralateral muscle movement [44]. Moreover, damage to mechanoreceptors limits the exertion of maximal torque by reducing the activation level of γ motor neurons, which affects bilateral muscle activity in unilateral joint injuries [45]. Considering the presence of bilateral AMI among people with CAI, using the strength of the unaffected limbs to represent uninjured strength may overestimate the recovery of ankle functions.

Our findings also revealed the presence of higher levels of AMI in the affected limbs than in the unaffected limbs among people with CAI. These have been supported by previous studies showing lower quadriceps CAR in the affected limbs compared to unaffected ones after anterior cruciate ligament reconstruction [29, 46]. Alterations in neural signals may occur in the affected limbs after ankle sprains, leading to adaptive changes in the muscles regulated by these signals over time. Changes in muscle fiber type [47] and fibrosis [48] have been observed after anterior cruciate ligament reconstruction, and these changes may be present after ankle sprains, persistently affecting muscle activity of peroneal muscles in the affected limbs.

This study has clinical significance. AMI limits the recovery of joint strength [15]. Approaches to improve nerve excitability (e.g., transcutaneous electrical nerve stimulation [49], cryotherapy [50], and vibration therapy [51]) should be included in ankle sprain rehabilitation to increase the activation of the inhibited motor neurons. Furthermore, even with unilateral ankle sprains, bilateral rehabilitation is necessary to eliminate or reduce the AMI in the unaffected limbs.

There are some limitations to this study. Superimposed stimulation may enhance the excitability of the antagonist’s muscle, thereby underestimating the CAR’s value since the “total force” would be reduced. Secondly, SIB technology only provides a transient stimulus and does not simulate natural movement. Finally, no X-rays were taken to check for bone morphology abnormalities in the fibula during recruitment, so the effect of bone morphology abnormalities in the fibula on muscle strength could not be clarified.

## Conclusions

AMI of peroneal muscles was observed bilaterally among people with CAI, and their affected limbs have higher AMI levels than the unaffected limbs. AMI may be a contributing factor to the decreased peroneal strength among people with CAI. Bilateral AMI rehabilitation should be performed even in those with unilateral CAI.

## Data Availability

Data are available upon request.

## Abbreviations

CAI: Chronic ankle instability
AMI: Arthrogenic muscle inhibition
CAR: Central activation ratio
MVIC: Maximal voluntary isometric contraction
SIB: Superimposed burst
ICC: Intra-class correlation coefficients
CI: confidence interval
